# The C-Terminal Domain of *Liquorilactobacillus nagelii* Dextransucrase Mediates the Production of Larger Dextrans Compared to *Liquorilactobacillus hordei*

**DOI:** 10.3390/gels8030171

**Published:** 2022-03-09

**Authors:** Julia Bechtner, Verena Hassler, Daniel Wefers, Matthias Ehrmann, Frank Jakob

**Affiliations:** 1 Department of Microbiology, Technical University Munich, 85354 Freising, Germany; 2 Institute of Chemistry, Food Chemistry—Functional Food, Martin-Luther-University Halle-Wittenberg, 06120 Halle, Germany; verena.hassler@chemie.uni-halle.de (V.H.); daniel.wefers@chemie.uni-halle.de (D.W.); 3 Department of Technical Microbiology, Technical University Munich, 85354 Freising, Germany; frank.jakob@tum.de

**Keywords:** dextransucrase, processivity, exopolysaccharide

## Abstract

Dextransucrases released by certain lactic acid bacteria form glucose polymers with predominantly α-1,6-linkages and may be exploited biotechnologically for the tailored production of polysaccharides with application potential. Despite releasing two closely related dextransucrases, previous studies showed that water kefir borne *Liquorilactobacillus* (*L.*) *hordei* TMW 1.1822 and *L. nagelii* TMW 1.1827 produce different amounts of polysaccharides with distinct particle sizes (molecular weight and radius of gyration) and molecular architectures. To investigate where these differences originate and thus to provide deeper insights into the functionally diverse nature of polysaccharide formation during water kefir fermentation, we constructed two variants of the *L. nagelii* dextransucrase—a full-length enzyme and a truncated variant, devoid of a C-terminal glucan-binding domain that reflects the domain architecture of the *L. hordei* dextransucrase—and applied them at various enzyme concentrations to form dextran over 24 h. The full-length enzyme exhibited a high activity, forming constant amounts of dextran until a four-fold dilution, whereas the truncated variant showed a gradual decrease in activity and dextran formation at an increasing dilution. The application of the full-length enzyme resulted in higher average particle sizes compared to the truncated variant. However, the dilution of the enzyme extracts also led to a slight increase in the average particle size in both enzymes. Neither the domain architecture nor the enzyme concentration had an impact on the structural architecture of the dextrans. The presented results thus suggest that the comparatively higher processivity of the *L. nagelii* dextransucrase is predominantly caused by the additional C-terminal glucan-binding domain, which is absent in the *L. hordei* dextransucrase. The average particle size may be influenced, to some extent, by the applied reaction conditions, whereas the structural architecture of the dextrans is most likely caused by differences in the amino acid sequence of the catalytic domain.

## 1. Introduction

Because polysaccharides alter the behavior of aqueous solutions, they are biotechnologically exploited to viscosify, chelate, emulsify, stabilize and retain water or to form films, membranes or gels [1]. Many polysaccharides of algae or plant origins, such as starch, cellulose, gum arabic and carrageenan, have a long history of industrial application [2,3]. Additionally, microbial polysaccharides have gained interest for application purposes, as they can be produced locally under controllable environmental conditions [4]. Herein, microbial polysaccharides may have the highest potential in industrial niches that demand a high degree of purity or specific characteristics of polysaccharides, such as in pharmaceuticals, cosmetics, foods or medicine [5]. According to their monomeric composition, microbial polysaccharides are distinguished into heteropolysaccharides (HePS), consisting of multiple types of monosaccharides, and homopolysaccharides (HoPS), exhibiting only a single type of monosaccharide [6]. HePS are produced intracellularly upon the use of nucleotide-activated sugar donors and are subsequently secreted by different pathways that mainly predetermine where the assembled polysaccharide will reside outside the cell, i.e., attached to the cell or released. Whereas the synthesis of HePS is rather complex, requiring several catalytic steps that are mediated by various enzymes [7,8], HoPS are often synthesized by only one enzyme, which is either attached to the cell surface or released into the surrounding milieu [9,10]. Belonging to this group of enzymes, glycoside hydrolase family 70 (GH70) enzymes represent a diverse group of enzymes that can produce large and often branched extracellular polysaccharides (EPS) on the basis of sucrose, starch or maltooligosaccharides [11]. So far, the glucansucrase GH70 subgroup has only been identified in lactic acid bacteria (LAB) of the genera *Streptococcus, Weissella* and *Oenococcus* and several genera of the *Lactobacillaceae* family [11,12]. Glucansucrases use sucrose as a substrate to transfer the glucose moiety to the non-reducing end of an acceptor substrate, which may be water (hydrolysis) or a growing oligo- or polysaccharide chain (transglycosylation), but other acceptor substrates are utilized as well [13]. By transglycosylation, these enzymes form α-glucans with different linkage types within the polysaccharide backbone and branches [14]. Besides the characteristic GH70 catalytic domain, glucansucrases exhibit one or more glucan-binding domain at varying positions [15]. Some of these enzymes possess an N-terminal signal peptide, an N-terminal variable domain of unknown function and/or a cell-wall anchor [9]. This variance in domain architecture ultimately leads to a high diversity in size of these enzymes, ranging between 120–200 kDa, with some exceptions [11,16]. While single amino acids within and outside the substrate- and acceptor-binding sites were already identified to determine the linkage type of the polysaccharide backbone and branch points [17,18,19,20], it is rather scarcely understood where differences in the side-chain length and polymer size originate. However, all of these factors influence the techno-functional properties of the formed polysaccharides. As such, the linkage type determines the monomer’s orientation and may thus lead to the intramolecular formation of hydrogen bridges and, therefore, to a reduced solubility in water, as is postulated for cellulose [21,22]. Moreover, the polymer size was shown to alter the rheological properties of glucans and fructans [23,24,25]. It is thus necessary to study the factors affecting the molecular and macromolecular properties of the polysaccharides formed by extracellular sucrases in order to lay the basis for the tailored production of such.

As an environment rich in sucrose, water kefir was shown to be an excellent reservoir for LAB encoding various glucansucrases [26,27] and thus for the study of native extracellular glucan formation. Whereas some glucansucrases are responsible for the production of the water-insoluble α-glucan, constituting the gelatinous biofilm matrix of the kefir granules [28], others form α-glucans with colloidal properties that lead to the natural turbidity of the drinkable phase of this traditional fermented beverage [29]. Thereby, *Lentilactobacillus hilgardii* was proposed to be the main producer of the kefir granule polysaccharide, which is a dextran-type glucan with mainly α-1,6-glycosidic linkages and elevated amounts of α-1,3-linkages and branches [27]. *Liquorilactobacillus* (*L.*) *hordei* and *L. nagelii,* both isolated from water kefir, encoded dextransucrases with identical substrate- and acceptor-binding subsites, but with only an ~76% overall amino acid sequence identity within their GH70 catalytic domains. Nonetheless, when compared to other known glucansucrases, the *L. hordei* and *L. nagelii* dextransucrases appeared to be closely related [30]. Both enzymes formed dextrans with cloud-forming properties, and a structural analysis of the polysaccharides showed a comparable degree of branching [29,30,31]. However, differences in the endo-dextranase liberated oligosaccharides were observed. These results strongly suggested that the two dextrans have a comparable structural composition (with regards to linkages) but different side chain lengths, and thus a different structural architecture. In addition, the native dextransucrases that were recovered as crude enzyme extracts in buffered cell suspensions of *L. hordei* and *L. nagelii* showed differences in their overall activity and transglycosylation activity, as well as the particle size of formed dextrans [30]. While lower enzyme concentrations and a putative β-fructosidase that is expressed and released in the presence of sucrose may have caused the lower activity measured in enzyme extracts of *L. hordei* [30,32], it remained unknown why *L. nagelii* produced dextrans with higher particle sizes and a different abundance of fine-structural elements than *L. hordei*. This knowledge gap, however, hinders a more detailed understanding of these enzymes to (i) produce tailored polysaccharides, (ii) exploit these microorganisms as starter cultures for in situ dextran formation in innovative food and beverage fermentations and (iii) understand the functionally diverse nature of polysaccharide formation during water kefir fermentation. Therefore, we investigated the most remarkable difference among the primary structure of both enzymes, which was previously shown to be the additional C-terminal glucan-binding domain of the *L. nagelii* dextransucrase [30], which ultimately folds into domain V [33].

## 2. Results

### 2.1. Expression of Dextransucrase Variants

**Figure 1:**
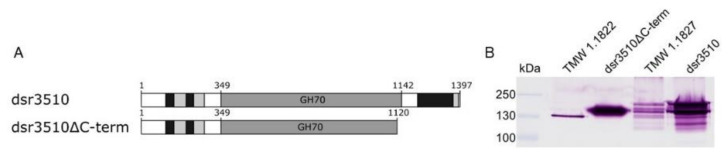
Domain architectures (primary sequences) of the heterologously expressed variants of the *L. nagelii* TMW 1.1827 dextransucrase. Black = glucan-binding domains, light gray = glucan-binding repeats, dark gray = GH70 catalytic domain (**A**). SDS-PAGE with subsequent activity staining of heterologously expressed dextransucrase variants compared to the native extracellular dextransucrases of *L. hordei* TMW 1.1822 and *L. nagelii* TMW 1.1827 (**B**).

The most distinctive difference between the native *L. hordei* TMW 1.1822 and *L. nagelii* TMW 1.1827 dextransucrases is the different constitution of domain V, particularly the additional C-terminal glucan-binding domain of the *L. nagelii* dextransucrase [30]. To investigate if differences in native extracellular dextran formation originate from that, the *L. nagelii* dextransucrase was heterologously expressed as either full length (dsr3510) or as a truncated variant (dsr3510ΔC-term) (Figure 1A).

As shown in Figure 1B, both variants were expressed and functional, as indicated by the activity staining. The truncated variant exhibited only one band, which was found at a larger size than the native extracellular *L. hordei* TMW 1.1822 dextransucrase (118.1 kDa), as it had a predicted molecular weight of 122.9 kDa. The full-length variant was found at the expected size range, as it had a predicted molecular weight of 155.2 kDa. It showed a band pattern comparable to the native extracellular dextransucrase of *L. nagelii* TMW 1.1827, which is the only GH70 enzyme predicted from the whole-genome sequence of this strain (Accession No. CP018180.1–CP018183.1) [30].

### 2.2. Characterization of the Native Extracellular and Heterologously Expressed Dextransucrase Variants

Both dextransucrase variants were examined for their overall activity at various pH and temperatures. As it was not possible to purify the heterologously expressed variants without a complete loss of activity, both variants were used as crude enzyme extracts. Therefore, the lysates of un-induced *E. coli* Rosetta cells were used as a control, but no activity against sucrose, fructose or glucose could be detected at the applied reaction conditions.

**Figure 2:**
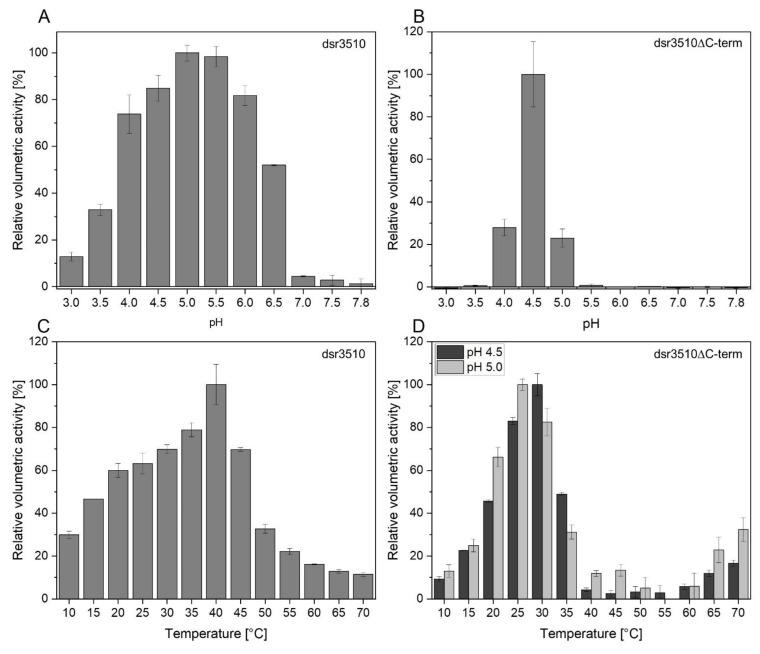
Relative volumetric activities ± SD of the heterologously expressed dextransucrase variants dsr3510 (**A**,**C**) and dsr3510ΔC-term (**B**,**D**) at various pH and temperatures. The pH optima were determined at 30 °C, whereas temperature optima were determined at pH 5.0 and pH 4.5 (dsr3510ΔC-term only). Volumetric activities were calculated relative to the highest overall activity, respectively.

As shown in Figure 2, the full-length dextransucrase variant exhibited activity over a broad range of pH and temperatures, whereas the truncated dextransucrase variant dsr3510ΔC-term showed measurable amounts of activity only at pH 4.0–5.0, with a clear optimum at pH 4.5. By contrast, the full-length variant dsr3510 showed maximum activity at pH 5.0–5.5. Below and above these pH values, the overall activity decreased rapidly, but was still detectable at extreme pH values of 3.0 and 7.8. The temperature optimum of the full-length variant dsr3510 was found to be at 40 °C. The truncated variant was tested at pH 4.5 and pH 5.0, as this enzyme was much less active at a non-optimum pH. At pH 4.5, its temperature optimum was 30 °C, whereas it was 25 °C at pH 5.0. An increase in activity could be detected for temperatures above 60 °C. Additionally, it could be shown that the overall activity of dsr3510 was increased by 21.4 ± 5.5 % when Ca^2+^ was added in the form of CaCl_2_. This was even more remarkable for the activity of the dsr3510ΔC-term, which increased by 50.7 ± 7.5 %. None of the other cations (Cu^2+^, Fe^2+^, Mg^2+^, Mn^2+^ or Na^+^) enhanced the activity of the heterologously expressed variants. 

All dextransucrase variants followed Michaelis–Menten kinetics. The heterologously expressed variants exhibited K_M_ values comparable to that of the native extracellular dextransucrase of *L. nagelii* TMW 1.1827, as listed in Table 1. Both the native *L. nagelii* dextransucrase and the full-length enzyme dsr3510 exhibited similar and high maximum reaction rates compared to the truncated variant.

### 2.3. Analysis of Dextrans Formed by the Native Extracellular and Heterologously Expressed Dextransucrase Variants

In addition to evaluating the role of the C-terminal glucan-binding domain of the *L. nagelii* dextransucrase, the influence of varying enzyme concentrations on the transglycosylation activity, amount of isolable dextran and structural architecture of the formed dextrans was tested. The obtained results were statistically analyzed, and the *p*-values are summarized in Table S1.

**Figure 3:**
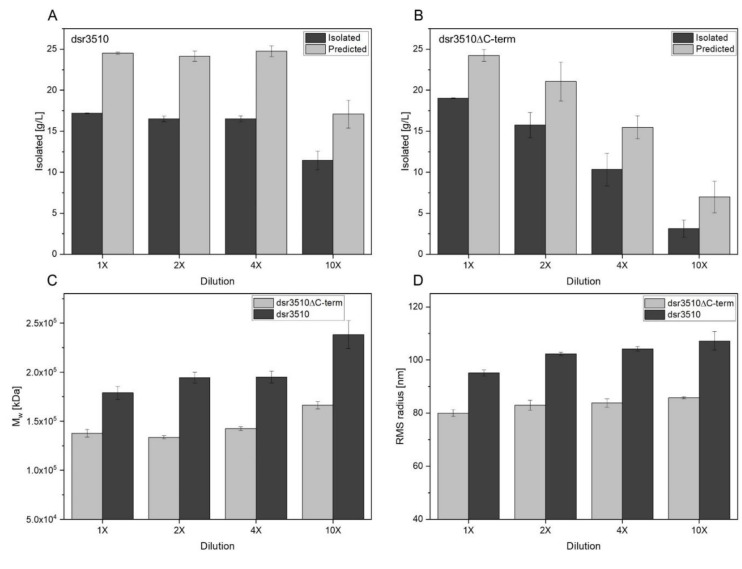
Isolated and predicted (=calculated on the basis of transglycosylation rates) amounts of dextrans produced by heterologously expressed variants dsr3510 (**A**) and dsr3510ΔC-term (**B**) for 24 h at 30 °C and pH 5.0, applying different enzyme concentrations. Particle sizes of the formed dextrans were determined by AF4-MALS-UV measurements with regard to their average molecular weights (M_w_, (**C**)) and root-mean-square (RMS) radii (**D**). Values are given ± SD.

The full-length enzyme dsr3510 produced constant amounts of isolable and predicted dextran (= calculated on the basis of transglycosylation activity) until a four-fold dilution of the enzyme extracts was reached (see Figure 3A,B). At a 10-fold dilution, the activity and isolated dextran amount decreased significantly (*p* = 0.004).

By contrast, the isolated and predicted amount of dextran produced by the truncated variant decreased gradually with an increasing dilution of the enzyme extract. The differences were tested as statistically significant among all concentrations (*p* < 0.05). Nonetheless, if not diluted, the amount of predicted dextrans of this enzyme variant was comparable to those of dsr3510 until a four-fold dilution, indicating a similar transglycosylation activity. By contrast, the amount of isolated dextran was slightly, but, yet, significantly, higher (*p* ≤ 0.001) when dextrans were produced with the undiluted dsr3510∆ C-term than with dsr3510, until a four-fold dilution.

The average root-mean-square (RMS) radii obtained from AF4-MALS-UV measurements ranged from 95.1–107.2 nm for the dsr3510 variant. The radii of the truncated variant dsr3510ΔC-term were consistently smaller at 80.0–85.8 nm, which was confirmed by statistical analyses (see Figure 3D). Regarding the enzyme concentration of dsr3510, only the undiluted enzyme produced significantly smaller dextrans compared to the other levels of dilution (*p* < 0.05). The difference in radius between dextrans produced by the truncated variant was only significantly different between the undiluted and the 10-fold diluted enzyme extracts (*p* = 0.003).

Similar results were obtained for the average molecular weight of the formed dextrans (see Figure 3C). Dsr3510 produced dextrans with gradually increasing M_w_ from 1.79–2.38 × 10^5^ kDa with an increasing enzyme dilution. The M_w_ of the dextrans produced by the truncated enzyme variant was consistently smaller, ranging from 1.38–1.66 × 10^5^ kDa. However, the difference between the 10-fold diluted truncated variant and the undiluted full-length enzyme was not statistically significant (*p* = 0.076).

**Figure 4:**
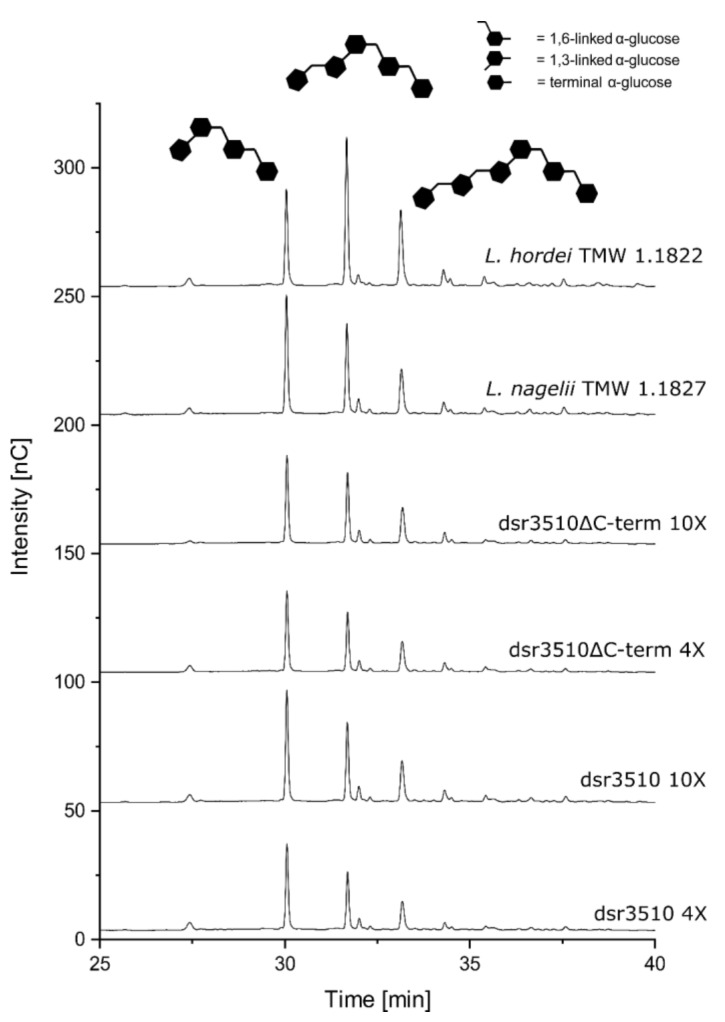
Endo-dextranase fingerprints of dextrans produced over 24 h at 30 °C by heterologously expressed dextransucrases dsr3510 and dsr3510ΔC-term that were applied at a 4-fold and 10-fold dilution of the enzyme extracts, respectively. For comparison, the dextrans produced by native extracellular dextransucrases of *L. hordei* TMW 1.1822 and *L. nagelii* TMW 1.1827 under equal reaction conditions were also subjected for this analysis.

In order to investigate the impact of the additional C-terminal glucan-binding domain of the *L. nagelii* dextransucrase on the dextran structural architecture, namely the side chain length, the dextrans were subjected to endo-dextranase digestion and a subsequent chromatographic analysis. As displayed in Figure 4, the two enzyme variants formed the same structural elements (at different enzyme concentrations). Furthermore, the relative abundance of the dextrans from both heterologously expressed variants was comparable to the native extracellular dextransucrase of *L. nagelii* TMW 1.1827, whereas the pattern of dextrans from native *L. hordei* TMW 1.1822 dextransucrase was clearly different. 

## 3. Discussion

Previous research on the fermentative production of dextrans [34], as well as native extracellular dextransucrases of *L. hordei* and *L. nagelii*, indicated that dextran formation during water kefir fermentation is functionally diverse, even among closely related species encoding highly similar dextransucrases [30]. This already started with the release of these enzymes, as *L. hordei* only released its dextransucrase in the presence of sucrose, despite intracellular accumulation at other conditions [30,32]. By contrast, *L. nagelii* released its dextransucrase independently of the presence of sucrose, if only in lower amounts than in the presence of sucrose [31]. Furthermore, a much higher amount of extracellular dextransucrase (activity) was detected in supernatants of *L. nagelii,* when both microorganisms were incubated at equal conditions, which consumed the whole sucrose within 10 min. By contrast, supernatants of *L. hordei* exhibited a distinctly lower amount of overall and transglycosylation activity, leading to the formation of lower amounts of isolable dextran over 24 h of incubation [30]. Therefore, it was unknown so far if the differences in the resulting polymer sizes and structural architectures were accountable to differences in the enzyme concentration, transglycosylation activity or differences in the enzyme’s domain architectures. We thus constructed two variants of the *L. nagelii* TMW 1.1827 dextransucrase and expressed them heterologously: a full-length variant (dsr3510) and a variant truncated by the C-terminal glucan-binding domain (dsr3510ΔC-term) that would otherwise be part of domain V in its three-dimensional structure [33]. Both dextransucrase variants were effectively expressed and functional, as shown from the SDS-PAGE analysis and subsequent activity staining. They exhibited their pH and temperature optima in the range of other GH70 enzymes [35,36,37]. However, the truncated variant was only active in a narrow range of pH values and temperatures compared to the full-length enzyme and had to be incubated for a longer time, as its overall activity was generally low. This indicates that the glucan-binding domain that eventually folds into domain V is necessary for a stable overall enzyme activity of the *L. nagelii* dextransucrase, including the integrity of the enzyme over a broad range of conditions. Furthermore, the presence of Ca^2+^ had a beneficial effect on the enzyme activity in both enzyme variants. A similar effect was already observed for other glucansucrases [36,38]. The calcium ion is proposed to form or stabilize the acceptor binding site of the enzyme [39]. However, Ca^2+^ improved the enzyme activity more drastically in the truncated variant than the full-length enzyme, implying that the truncated variant profits more from a stabilizing effect of the metal ion. This is in accordance with the hypothesis inferred above, proposing that the enzymatic activity of the truncated variant suffers from a reduced stability due to the lack of the C-terminal domain. Surprisingly, the truncated variant showed an increase in activity at temperatures above 60 °C. As enzymes of mesophilic bacteria are known to be sensitive to heat denaturation [40,41], this may rather be an unspecific reaction. Furthermore, both variants followed Michaelis–Menten kinetics, as was already shown for the native extracellular dextransucrase of *L. nagelii* TMW 1.1827 [31]. The K_M_ values were within the range of other characterized glucansucrases [37,42,43] and were comparable to the K_M_ of the native extracellular dextransucrase of *L. nagelii* TMW 1.1827 [31]. However, the v_max_ was quite versatile for both variants and was distinctly lower than the v_max_ of the undiluted native *L. nagelii* dextransucrase. Nonetheless, it was shown that, for this enzyme, the v_max_ decreases linearly with increasing dilution [31] and may thus indicate a lower dextransucrase concentration at the applied conditions within the crude enzyme extracts containing the heterologously expressed variants.

As experiments on the native extracellular dextran formation of *L. hordei* TMW 1.1822 and *L. nagelii* TMW 1.1827 were conducted at pH 5.0 and 30 °C for 24 h [30], equal conditions were also applied to form dextrans with the heterologously expressed variants. The full-length enzyme exhibited high transglycosylation rates and isolated amounts of dextran that stayed constant until a four-fold dilution of the enzyme extract. At a 10-fold dilution of the enzyme extract, the transglycosylation activity decreased, along with a decrease in isolated dextran. A comparable pattern was observed for the native extracellular dextransucrase of *L. nagelii* TMW 1.1827 in a previous study [31]. As shown for the native extracellular dextransucrase of *L. nagelii* TMW 1.1827, this indicates that fewer enzymes can form the same amount of dextran, but more slowly and only until a critical enzyme concentration is reached. By contrast, the truncated variant only exhibited a comparable amount of activity and a slightly higher amount of isolable dextran as dsr3510 when it was applied undiluted. The diluted enzyme extracts of the truncated variant, however, showed a gradual decrease in dextransucrase activity and isolable dextran with an increasing dilution, indicating that the amount of dextransucrase is much lower than in the enzyme extracts containing full-length dsr3510.

Both enzyme variants had similarities in that the RMS radii and molecular weights of the isolated dextrans increased slightly with an increasing dilution; however, only with a significant increase in particle size at a 10-fold dilution. This shows that a higher transglycosylation rate is not necessarily correlated with the degree of polymerization and rather reflects the productivity of the enzyme. Therefore, the average polymer size may only be influenced to a minor extent by the applied enzyme concentration. However, it was hypothesized before that supernatants of *L. hordei* TMW 1.1822 exhibit much lower dextransucrase amounts than supernatants of *L. nagelii* TMW 1.1827 and would therefore produce dextrans with higher molecular weights and RMS radii, whereas our previous study demonstrated the opposite [30]. Moreover, the average molecular weights and RMS radii of the dextrans produced by the heterologously expressed full-length variant were consistently higher than those of the dextrans produced by the truncated variant. While some differences in polymer size are indeed explainable by varying enzyme concentrations, these results clearly demonstrate that the higher processivity of the native *L. nagelii* dextransucrase is caused by the C-terminal glucan-binding domain that is absent in the *L. hordei* dextransucrase. This is similar to the results of Claverie et al. [44], who showed that domain V, which is altered by the presence or absence of a C-terminal glucan-binding domain in the present study, mediates processivity in GH70 enzymes. These results may furthermore explain why *L. nagelii* TMW 1.1827 produces larger dextrans than *L. hordei* TMW 1.1822, even under fermentative conditions, despite equal incubation conditions [34], hinting at similar processes during water kefir fermentation.

However, the additional glucan-binding domain and the potentially resulting differences in protein folding did not influence the structural architecture of the synthesized dextran, as portions of different oligosaccharides liberated by endo-dextranase digestions were comparable for the full-length enzyme and the truncated variant. These fine structures, namely the side-chain length, were, furthermore, not influenced by the applied enzyme concentration. Despite being not directly involved in substrate- or acceptor-binding, several more distantly located amino acid residues have been identified to influence the linkage pattern of synthesized dextrans [45,46]. These residues may either be directly involved in shaping the active site or influence those residues by steric interactions [47]. The *L. hordei* and *L. nagelii* dextransucrases were previously shown to be identical regarding substrate and acceptor binding subsites; however, only ~76 % of the amino acids within the GH70 catalytic domain were identical [30]. Therefore, differences in the amino acid sequence of the catalytic domain most likely led to the observed differences in the molecular architecture of the dextrans synthesized by native dextransucrases of *L. hordei* and *L. nagelii*. 

In summary, it could be shown that the differences in the polymer size of the dextrans formed by native extracellular dextransucrases of *L. nagelii* and *L. hordei* are predominantly a result of different domain architectures of the closely related enzymes. In particular, this results from differences in domain V, which includes an additional C-terminal glucan-binding domain within the *L. nagelii* dextransucrase, leading to a higher processivity of this enzyme compared to the one released by *L. hordei*. Nonetheless, our results indicate that the average molecular weight and RMS radius may be adjusted to a minor extent by different dextransucrase concentrations. However, the structural architecture was independent of differences in domain V or the enzyme concentration, suggesting that the amino acid sequence of the catalytic domain is responsible for different proportions of structural elements liberated by endo-dextranase. Our results thus give new insights into the functionally diverse nature of α-glucan formation during water kefir fermentation, even in highly similar glucansucrases, and lay the basis for sophisticated applications of these enzymes or their corresponding microorganisms in innovative food and beverage fermentations.

## 4. Materials and Methods

### 4.1. Strains, Media and Growth Conditions

*L. hordei* TMW 1.1822 and *L. nagelii* TMW 1.1827, both isolated from water kefir, were cultivated statically in closed vessels at 30 °C in 15 mL of modified liquid MRS medium (10 g/L soy peptone, 10 g/L meat extract, 5 g/L yeast extract, 20 g/L glucose, 1 g/L Tween80, 2 g/L dipotassium phosphate, 5 g/L sodium acetate, 2 g/L di-ammonium citrate, 0.2 g/L magnesium sulfate, 0.05 g/L manganese sulfate, pH 6.2). For cloning and heterologous expression, *Escherichia* (*E.*) *coli* DH5α, which carried the pBAD-*Myc*-HisA plasmid, and *E. coli* Rosetta (for heterologous expression) were used. Both were cultivated in lysogeny broth (10 g/L tryptone, 5 g/L yeast extract, 5 g/L sodium chloride, pH 7.2) under oxic conditions at 37 °C unless stated otherwise. For plasmid maintenance, 100 µg/mL ampicillin (amp100) and, additionally, 68 µg/mL chloramphenicol (cmp68) for the cultivation of *E. coli* Rosetta were added to the medium. For solid media, 15 g/L of agar was added.

### 4.2. Molecular Techniques and Plasmid Construction

Cloning, transformation of *E. coli* Rosetta and DNA manipulations were performed according to the general techniques described by Sambrook et al. [48]. The identified dextransucrase gene of *L. nagelii* TMW 1.1827 (dsr3510) and a truncated variant of the same dextransucrase (dsr3510ΔC-term), shortened by the deletion of its C-terminal glucan-binding domain (see Figure 1), were cloned into pBAD/*Myc*-HisA expression vectors (Invitrogen, Carlsbad, CA, USA) that code for a C-terminal 6× histidine tag for subsequent purification of the expressed proteins. Genomic DNA of *L. nagelii* TMW 1.1827 was isolated using the E.Z.N.A.™ Bacterial DNA Kit (Omega Bio-Tek Inc., Norcross, GA, USA) according to the manufacturer’s instructions, but with a prolonged incubation time of 2 h for cell lysis. Plasmid DNA of *E. coli* DH5α was isolated using the QIAprep Spin Miniprep Kit (Qiagen, Hilden, Germany) according to the manufacturer’s instructions. Primers used for the amplification of the dextransucrase variants are listed in Supplementary Table S2 and amplification was performed by PCR using the Phusion High Fidelity DNA Polymerase Kit (New England BioLabs Inc., Ipswich, MA, USA) according to the manufacturer’s instructions, applying a temperature gradient of 65 ± 5 °C for primer annealing. Subsequently, both PCR products, as well as the vector, were cut using the FastDigest™ restriction enzymes *XhoI* and *Bsp119I* (ThermoFisher Scientific, Waltham, MA, USA) according to manufacturer’s instructions. The vector DNA was additionally treated with shrimp alkaline phosphatase (1 u/µL, New England BioLabs Inc., Ipswich, MA, USA) by direct addition of the enzyme to the restriction digest reaction. DNA samples were purified using the E.Z.N.A.™ Cycle Pure Kit (Omega Bio-tek Inc., Norcross, GA, USA) according to the manufacturer’s instructions. Then, both dextransucrase variants were ligated into the cut pBAD/*Myc*-HisA vector using the T4 DNA Ligase Kit (ThermoFisher Scientific, Waltham, MA, USA) according to manufacturer’s instructions. Subsequently, 100 µL of *E. coli* Rosetta that was prepared as chemically competent by the rubidium chloride method [49] was mixed with 10 µL of construct DNA (0.01 µg/µL), respectively. Transformation was carried out by incubation on ice for 20 min, followed by heat shocking for 90 s at 42 °C. After incubation on ice for another 2 min, 4 volumes of lysogeny broth were added. A total of 100 µL of each transformation mixture was plated on solid lysogeny broth media (amp100, cmp68) and incubated overnight at 37 °C. To control for correct amplification and insertion of the dextransucrases, plasmid DNA of both variants was isolated as described above and sent to Eurofins Genomics (Ebersberg, Germany) for SupremeRun Sanger sequencing using the primers listed in Supplementary Table S2.

### 4.3. Isolation and Verification of the Dextransucrases

The isolation of the native dextransucrases of *L. hordei* TMW 1.1822 and *L. nagelii* TMW 1.1827 was performed as described before [30]. Briefly, cells were pre-cultured for 48 h to inoculate 45 mL of fresh medium to an OD_590 nm_ of 0.1. After 24 h of incubation at 30 °C, cells were harvested by centrifugation (5000× *g*, 4 °C, 10 min) and subsequently resuspended in 15 mL citrate-phosphate buffer (0.05 M citrate, 0.1 M disodium phosphate, 0.05 M sucrose, pH 6.5). After incubation for 3 h at 30 °C, cells were removed by centrifugation (5000× *g*, 4 °C, 10 min) and supernatants were subsequently filtered sterile (0.2 µm nylon filters, Phenomenex, Germany).

The dextransucrase variants transformed into *E. coli* Rosetta, respectively, were yielded by cultivation in 100 mL lysogeny broth (amp100, cmp68) to an OD_590_ of approx. 0.5–0.6 at 37 °C and 200 rpm. Expression was induced by L-arabinose addition to a final concentration of 0.2 % (*w*/*v*) and cultures were subsequently incubated overnight at 16 °C and 150 rpm. The cells were harvested by centrifugation (5000× *g*, 4 °C, 10 min) and resuspended in 5 mL of citrate-phosphate buffer (0.05 M citrate, 0.1 disodium phosphate, pH 5.0), and cell lysis was performed by sonification on ice. Cell debris was removed afterwards by centrifugation (10,000× *g*, 4 °C, 15 min). Protein concentrations were measured using the Coomassie (Bradford) Protein Assay Kit (ThermoFisher Scientific, Waltham, MA, USA) according to the manufacturer’s instructions on the standard microplate procedure.

To verify the presence of the native and heterologously expressed dextransucrase variants, all samples were applied for vertical sodium dodecyl sulfate polyacrylamide gel electrophoresis (SDS-PAGE) carried out in a Mini-PROTEAN^®^ Tetra Cell Electrophoresis System (Bio-Rad laboratories, Hercules, CA, USA) as described before [31]. A separation gel (10% (*w*/*v*)) with a stacking gel (4% (*w*/*v*)) were used. Protein samples were diluted in 2 × Laemmli buffer (Sigma-Aldrich, St. Louis, MO, USA) and denatured at 90 °C for 10 min prior to application. Separation was initially started at 100 V for 10 min and continued at 150 V for 60 min using a Power Pack 3000 unit (Bio-Rad laboratories, Hercules, CA, USA). Selective visualization of the dextransucrases was carried out by activity staining, as described in Bechtner et al. [30]. In brief, gels were washed three times for 10 min in sodium acetate buffer (20 mM, 0.3 mM CaCl_2_, 0.1% Tween 80, pH 5.4) at 4 °C and incubated in the same buffer, containing 5% (*w*/*v*) sucrose overnight at 30 °C. The gels were washed for 30 min in an aqueous solution containing 50% (*v*/*v*) methanol and 10% (*v*/*v*) acetic acid. After another washing step of 30 min in deionized water (dH_2_O), formed dextrans were oxidized by incubation in periodic acid solution (1% (*w*/*v*) periodic acid, 3% (*v*/*v*) acetic acid) for 45 min. Subsequently, gels were washed again in dH_2_O for 1 h. Finally, dextrans were stained by Schiff’s reagent (Sigma-Aldrich, St. Louis, MO, USA) until discrete magenta bands appeared. The gels were washed in dH_2_O for 5 min. All steps were conducted at room temperature, if not stated otherwise.

### 4.4. Activity Measurements of Dextransucrases

The optimum pH and temperature of the heterologously expressed dextransucrases were determined in citrate-phosphate buffer (0.05 M citrate, 0.1 M disodium phosphate) supplemented with 0.2 M sucrose. The pH optima were determined in a range between pH 3.0 and 7.8 at 30 °C and temperature optima were determined in a range between 10 to 70 °C at pH 5.0, except for the temperature optimum of the variant dsr3510ΔC-term, which was additionally determined at pH 4.5. Additionally, the influence of cations was tested by the addition of 1 mM CaCl_2_, CuCl_2_, FeCl_2_, MgCl_2_, MnCl_2_ or NaCl, respectively. For this purpose, the citrate-phosphate buffer was replaced by sodium acetate buffer (0.05 M, pH 5.0). The assays were incubated for 60 min (dsr3510) or 180 min (dsr3510ΔC-term) and the reactions were stopped by the addition of 5 µL of a 2.5 M sodium hydroxide solution. Experiments on the heterologously expressed dextransucrase variants were conducted using the crude enzyme extracts, with an overall protein concentration adjusted to 200 µg/mL, if not stated otherwise. The Michaelis constants (K_M_) and maximum reaction rates (v_max_) were determined in sodium acetate buffer (0.05 M, 1 mM CaCl_2_, pH 5.0) at 30 °C using sucrose concentrations from 1.56–500 mM. The dsr3510 variant was added at an overall protein concentration of 500 µg/mL and incubated for 10 min, whereas dsr3510ΔC-term was added at an overall protein concentration of 2500 µg/mL and incubated for 60 min. As negative control, all mixtures were additionally prepared without the addition of enzyme extract. Additionally, cell lysates of un-induced *E. coli* Rosetta cultures were tested for activity on sucrose, glucose and fructose. All reactions were performed as triplicates. Subsequently, glucose and fructose concentrations were quantified by the D-Fructose/D-Glucose Assay Kit (Megazyme, Wicklow, Ireland) according to the manufacturer’s instructions on the microplate procedure, but with the addition of 10 µL of solution 3 and solution 4, respectively. The overall activities of the enzymes were calculated on the basis of released fructose amounts, whereas transglycosylation rates were calculated by the difference between released fructose and glucose. The transglycosylation rates were subsequently used to predict the possible amount of formed dextran.

K_M_ and v_max_ were calculated on the basis of overall enzyme activities using the “Enzyme kinetics” plugin tool within the OriginPro software (v. 9.7, OriginLab Corporation, Northampten, MA, USA) under default settings for Michaelis–Menten kinetics.

### 4.5. Enzymatic Dextran Formation

Dextran formation by the native extracellular dextransucrases was performed as described before [30]. The heterologously expressed variants were recovered in sodium acetate buffer (0.05 M, 1 mM CaCl_2_, pH 5.0) and adjusted to an overall protein concentration of 1000 µg/mL (=undiluted = 1X) in the same buffer. The enzyme solutions were additionally diluted 2-fold, 4-fold and 10-fold in the same buffer. Subsequently, one volume of each dilution was mixed with an equal volume of sodium acetate buffer supplemented with 0.4 M sucrose. At the end of each reaction, a 100 µL sample was taken and stopped by the addition of 10 µL of a 2.5 M sodium hydroxide solution. All reactions were carried out as triplicates and were incubated at 30 °C for 24 h. The samples taken afterwards were subsequently subjected for glucose and fructose quantification.

The rest of each reaction mixture was then used to purify the formed dextrans. The samples were treated with trichloroacetic acid (10% (*w*/*v*)) for 10 min on ice and subsequent centrifugation (15,000× *g*, 4 °C, 20 min) for protein precipitation. Subsequently, samples were dialyzed against dH_2_O for 2 days at 4 °C using MEMBRA-CELL dialysis tubes (SERVA, Heidelberg, Germany) with a molecular weight cut-off of 3.5 kDa. The purified polysaccharides were subsequently lyophilized and quantified by weighing.

### 4.6. Determination of Molecular and Macromolecular Structures of the Formed Dextrans

The structural architecture of the dextrans produced by the heterologously expressed variants that were diluted 4-fold and 10-fold, respectively, was analyzed by high-performance anion exchange chromatography with pulsed amperometric detection (HPAEC-PAD) after endo-dextranase (E.C. 3.2.1.11, from *Chaetomium* sp., Megazyme, Ireland, 5 U/mg polysaccharide) digestion for 24 h at 40 °C. Chromatographic analysis of the resulting oligosaccharides was carried out as described previously [50]. As a control, the dextrans produced by the undiluted native dextransucrases of *L. hordei* TMW 1.1822 and *L. nagelii* TMW 1.1827 were analyzed for comparison.

Molar masses and radii of the enzymatically formed dextrans were determined by asymmetric flow field-flow fractionation (AF4) (Wyatt Technology, Dernbach, Germany) coupled to multi-angle laser light scattering (MALS) (Dawn Heleos II, Wyatt Technology, Dernbach, Germany) and UV detection as quantitative detector (Dionex), as described previously [30]. First, lyophilized dextrans were redissolved in dH_2_O at a concentration of 0.1 mg/mL. A total of 100 µL of each sample was automatically injected to the AF4 channel, equipped with a 10 kDa regenerated cellulose membrane (Superon GmbH, Dernbach, Germany), using an aqueous solution with 50 mM NaNO_3_ as eluent. The injection flow was 0.2 mL/min, the focus flow was 1.5 mL/min and the cross flow was a linear gradient from 3.0 to 0.1 mL/min within 10 min. Then, the cross flow was kept at 0.1 mL/min for 15 min and subsequently set to 0 mL/min for the elution of remaining particles. The analysis of the MALS signals was performed using ASTRA software (v. 6.1, Wyatt Technology, Dernbach, Germany). RMS radii were determined in particle mode using the Berry model (best fit, suitable for M_w_ > 1 × 10^6^ Da). Molar masses were absolutely determined applying a refractive index increment (*dn*/*dc*) of 0.1423 mL/g for dextrans [51]. Specific UV extinction coefficients, necessary for concentration determination, were measured at 400 nm and calculated for each produced dextran as described by Ua-Arak et al. [52].

### 4.7. Statistical Analysis and Data Visualization

The amounts of isolated and predicted dextrans produced by both enzyme variants at different concentrations, as well as molecular weights and radii of these dextrans, were compared statistically using the two-sample t-test procedure provided by the Perseus software package (v. 1.6.14.0) [53]. Data visualization was performed using OriginPro software (v. 9.7, OriginLab Corporation, Northampton, MA, USA).

## Figures and Tables

**Table 1:** Michaelis constants (K_M_) and maximum reaction rates (v_max_) of the native extracellular dextransucrase of *L. nagelii* TMW 1.1827, as well as the heterologously expressed dextransucrase variants dsr3510 and dsr3510ΔC-term at pH 5.0 and 30 °C. Reaction mixtures of heterologously expressed variants contained 1 mM CaCl_2_. Values are given ± SD. * Adapted from Bechtner et al. [31].

Dextransucrase Variant	K_M_ [mM]	v_max_ [mmol/min × L^−1^]
*L. nagelii* TMW 1.1827	12.99 ± 3.74 *	1.03 ± 0.07 *
dsr3510	10.97 ± 1.56	0.200 ± 0.006
dsr3510ΔC-term	12.57 ± 0.73	0.089 ± 0.002

## Data Availability

Not applicable.

## Supplementary Materials

The following supporting information can be downloaded at: https://www.mdpi.com/article/10.3390/gels8030171/s1, Table S1: Table of *p*-values representing statistical significance regarding isolated and predicted amounts of dextran and respective average molecular weights (M_w_) and average rms radii as obtained from comparison of dextrans produced by distinct amounts of enzyme extracts of the heterologously expressed dextransucrase variants dsr3510 and dsr3510ΔC-term. Light gray = 0.05 > *p* > 0.01; gray = 0.01 ≥ *p* > 0.001; dark gray = *p* ≤ 0.001. Table S2: Primers used for the cloning and sequencing of the two dextransucrase variants dsr3510 and dsr3510ΔC-term.
