# Supplementary material for: The C-Terminal Domain of Liquorilactobacillus nagelii Dextransucrase Mediates the Production of Larger Dextrans Compared to Liquorilactobacillus hordei
Source: Gels. 2022 Mar 9;8(3):171. doi: 10.3390/gels8030171 (PMC8954249; doi:10.3390/gels8030171)
Supplement: Supplementary file 1 [file gels-08-00171-s001.zip › gels-1610564-supplementary.pdf]

Supplementary Materials

The C-terminal Domain of *Liquorilactobacillus nagelii* Dextransucrase Mediates the Production of Larger Dextrans Compared to *Liquorilactobacillus hordei*

Julia Bechtner[1*], Verena Hassler[2], Daniel Wefers[2], Matthias Ehrmann[1*] and Frank Jakob[3]

Table S1. Table of *p*-values representing statistical significance regarding isolated and predicted amounts of dextran and respective average molecular weights (M_w) and average rms radii as obtained from comparison of dextrans produced by distinct amounts of enzyme extracts of the heterologously expressed dextransucrase variants dsr3510 and dsr3510ΔC-term. Light gray = $0.05 > p > 0.01$; gray = $0.01 \geq p > 0.001$; dark gray = $p \leq 0.001$.

**Dextran isolated**	**dsr3510ΔC-term 1X**	**dsr3510ΔC-term 2X**	**dsr3510ΔC-term 4X**	**dsr3510ΔC-term 10X**	**dsr3510 1X**	**dsr3510 2X**	**dsr3510 4X**	**dsr3510 10X**
**dsr3510ΔC-term 1X**	-	0.039	0.003	<0.001	<0.001	<0.001	<0.001	<0.001
**dsr3510ΔC-term 2X**	0.039	-	0.038	0.001	0.254	0.527	0.529	0.033
**dsr3510ΔC-term 4X**	0.003	0.038	-	0.011	0.008	0.012	0.012	0.528
**dsr3510ΔC-term 10X**	<0.001	0.001	0.011	-	<0.001	<0.001	<0.001	0.002
**dsr3510 1X**	<0.001	0.254	0.008	<0.001	-	0.048	0.066	0.002
**dsr3510 2X**	<0.001	0.527	0.012	<0.001	0.048	-	1.000	0.004
**dsr3510 4X**	0.001	0.529	0.012	<0.001	0.066	1.000	-	0.004
**dsr3510 10X**	0.001	0.033	0.528	0.002	0.002	0.004	0.004	-
**Dextran predicted**	**dsr3510ΔC-term 1X**	**dsr3510ΔC-term 2X**	**dsr3510ΔC-term 4X**	**dsr3510ΔC-term 10X**	**dsr3510 1X**	**dsr3510 2X**	**dsr3510 4X**	**dsr3510 10X**
**dsr3510ΔC-term 1X**	-	0.142	0.001	<0.001	0.644	0.880	0.510	0.005
**dsr3510ΔC-term 2X**	0.142	-	0.046	0.003	0.108	0.149	0.101	0.126
**dsr3510ΔC-term 4X**	0.001	0.046	-	0.007	0.001	0.001	0.001	0.361
**dsr3510ΔC-term 10X**	<0.001	0.003	0.007	-	<0.001	<0.001	<0.001	0.005
**dsr3510 1X**	0.644	0.108	0.001	<0.001	-	0.457	0.640	0.004
**dsr3510 2X**	0.880	0.149	0.001	<0.001	0.457	-	0.397	0.005
**dsr3510 4X**	0.510	0.101	0.001	<0.001	0.640	0.397	-	0.004
**dsr3510 10X**	0.005	0.126	0.361	0.005	0.004	0.005	0.004	-
**RMS radii**	**dsr3510ΔC-term 1X**	**dsr3510ΔC-term 2X**	**dsr3510ΔC-term 4X**	**dsr3510ΔC-term 10X**	**dsr3510 1X**	**dsr3510 2X**	**dsr3510 4X**	**dsr3510 10X**
**dsr3510ΔC-term 1X**	-	0.136	0.051	0.003	<0.001	<0.001	<0.001	<0.001
**dsr3510ΔC-term 2X**	0.136	-	0.616	0.106	0.002	<0.001	<0.001	0.001
**dsr3510ΔC-term 4X**	0.051	0.616	-	0.173	0.001	<0.001	<0.001	0.001

**dsr3510ΔC-term 10X**	0.003	0.106	0.173	-	<0.001	<0.001	<0.001	0.001
**dsr3510 1X**	<0.001	0.002	0.001	<0.001	-	0.002	0.001	0.010
**dsr3510 2X**	<0.001	<0.001	<0.001	<0.001	0.002	-	0.069	0.122
**dsr3510 4X**	<0.001	<0.001	<0.001	<0.001	0.001	0.069	-	0.304
**dsr3510 10X**	<0.001	0.001	0.001	0.001	0.010	0.122	0.304	-
**M_w**	**dsr3510ΔC-term 1X**	**dsr3510ΔC-term 2X**	**dsr3510ΔC-term 4X**	**dsr3510ΔC-term 10X**	**dsr3510 1X**	**dsr3510 2X**	**dsr3510 4X**	**dsr3510 10X**
**dsr3510ΔC-term 1X**	-	0.234	0.186	0.002	0.002	<0.001	<0.001	0.001
**dsr3510ΔC-term 2X**	0.234	-	0.007	<0.001	0.001	<0.001	<0.001	<0.001
**dsr3510ΔC-term 4X**	0.186	0.007	-	0.001	0.002	<0.001	<0.001	0.001
**dsr3510ΔC-term 10X**	0.002	<0.001	0.001	-	0.076	0.004	0.005	0.002
**dsr3510 1X**	0.002	0.001	0.002	0.076	-	0.065	0.063	0.006
**dsr3510 2X**	<0.001	<0.001	<0.001	0.004	0.065	-	0.908	0.015
**dsr3510 4X**	<0.001	<0.001	<0.001	0.005	0.063	0.908	-	0.017
**dsr3510 10X**	0.001	<0.001	0.001	0.002	0.006	0.015	0.017	-

Table S2. Primers used for the cloning and sequencing of the two dextransucrase variants dsr3510 and dsr3510ΔC-term.

Primer	Sequence (5′→3′)	Application
Nag-forward	GCCTCGAGAGATTCAACACCACAAAATG	Primer for cloning of both variants
Nag-complete-reverse	GCTTCGAAGCAAGTTTTCTACCGGTTTTAG	Primer for cloning of complete dextransucrase dsr3510
Nag-truncated-reverse	GCTTCGAAGCATTATCGTCACTACGTAAAAC	Primer for cloning of dsr3510ΔC-term dextransucrase
Nag-1-Fwd	CGGATCCTACCTGACGCTTT	Sequencing of constructs
Nag-2-Fwd	GGTGAGTACGAAAAAGTTGGCG	Sequencing of constructs
Nag-3-Fwd	AACTGGTTGCGTCAGATTATGC	Sequencing of constructs
Nag-4-Fwd	CAAAAGGCAATTCAAGCAGCCA	Sequencing of constructs
Nag-5-Fwd	GCTAACCCGGATGTAACTGGA	Sequencing of complete dextransucrase construct
Nag-6-Fwd	CTTGGTCGCGGTAGCGATTA	Sequencing of complete dextransucrase construct
Nag-7-Fwd	TGGCTGGCAGTATATTAGCG	Sequencing of complete dextransucrase construct
Nag-8-Fwd	ATTGCTGATTGGGTGCCGGA	Sequencing of truncated dextransucrase construct
Nag-9-Fwd	CTGATGACAATGCTCCGATTGC	Sequencing of truncated dextransucrase construct
